# A low-carbohydrate, ketogenic diet to treat type 2 diabetes

**DOI:** 10.1186/1743-7075-2-34

**Published:** 2005-12-01

**Authors:** William S Yancy, Marjorie Foy, Allison M Chalecki, Mary C Vernon, Eric C Westman

**Affiliations:** 1 Center for Health Services Research in Primary Care, Department of Veterans' Affairs Medical Center (152), 508 Fulton Street, Durham, NC, USA 27705; 2 Department of Medicine, Duke University Medical Center, Durham, NC, USA; 3 Private Bariatric and Family Practice, and Clinical Faculty, University of Kansas School of Medicine, Lawrence, KS, USA

## Abstract

**Background:**

The low-carbohydrate, ketogenic diet (LCKD) may be effective for improving glycemia and reducing medications in patients with type 2 diabetes.

**Methods:**

From an outpatient clinic, we recruited 28 overweight participants with type 2 diabetes for a 16-week single-arm pilot diet intervention trial. We provided LCKD counseling, with an initial goal of <20 g carbohydrate/day, while reducing diabetes medication dosages at diet initiation. Participants returned every other week for measurements, counseling, and further medication adjustment. The primary outcome was hemoglobin A_1c_.

**Results:**

Twenty-one of the 28 participants who were enrolled completed the study. Twenty participants were men; 13 were White, 8 were African-American. The mean [± SD] age was 56.0 ± 7.9 years and BMI was 42.2 ± 5.8 kg/m^2^. Hemoglobin A_1c _decreased by 16% from 7.5 ± 1.4% to 6.3 ± 1.0% (p < 0.001) from baseline to week 16. Diabetes medications were discontinued in 7 participants, reduced in 10 participants, and unchanged in 4 participants. The mean body weight decreased by 6.6% from 131.4 ± 18.3 kg to 122.7 ± 18.9 kg (p < 0.001). In linear regression analyses, weight change at 16 weeks did not predict change in hemoglobin A_1c_. Fasting serum triglyceride decreased 42% from 2.69 ± 2.87 mmol/L to 1.57 ± 1.38 mmol/L (p = 0.001) while other serum lipid measurements did not change significantly.

**Conclusion:**

The LCKD improved glycemic control in patients with type 2 diabetes such that diabetes medications were discontinued or reduced in most participants. Because the LCKD can be very effective at lowering blood glucose, patients on diabetes medication who use this diet should be under close medical supervision or capable of adjusting their medication.

## Background

Prior to the advent of exogenous insulin for the treatment of diabetes mellitus in the 1920's, the mainstay of therapy was dietary modification. Diet recommendations in that era were aimed at controlling glycemia (actually, glycosuria) and were dramatically different from current low-fat, high-carbohydrate dietary recommendations for patients with diabetes [1,2]. For example, the Dr. Elliot Joslin Diabetic Diet in 1923 consisted of "meats, poultry, game, fish, clear soups, gelatin, eggs, butter, olive oil, coffee, tea" and contained approximately 5% of energy from carbohydrates, 20% from protein, and 75% from fat [3]. A similar diet was advocated by Dr. Frederick Allen of the same era [4].

Recently, four studies have re-examined the effect of carbohydrate restriction on type 2 diabetes. One outpatient study enrolled 54 participants with type 2 diabetes (out of 132 total participants) and found that hemoglobin A_1c _improved to a greater degree over one year with a low-carbohydrate diet compared with a low-fat, calorie-restricted diet [5,6]. Another study enrolled 8 men with type 2 diabetes in a 5-week crossover outpatient feeding study that tested similar diets [7]. The participants had greater improvement in glycohemoglobin while on the low-carbohydrate diet than when on a eucaloric low-fat diet. The third study was an inpatient feeding study in 10 participants with type 2 diabetes [8]. After only 14 days, hemoglobin A_1c _improved from 7.3% to 6.8%. In the fourth study, 16 participants with type 2 diabetes who followed a 20% carbohydrate diet had improvement of hemoglobin A_1c _from 8.0% to 6.6% over 24 weeks [9]. Only these latter three studies targeted glycemic control as a goal, and two of these were intensely-monitored efficacy studies in which all food was provided to participants for the duration of the study [7,8]. Three of the studies [6,8,9] mentioned that diabetic medications were adjusted but only one of them provided detailed information regarding these adjustments [9]. This information is critical for patients on medication for diabetes who initiate a low-carbohydrate diet because of the potential for adverse effects resulting from hypoglycemia.

The purpose of this study was to evaluate the effects of a low-carbohydrate, ketogenic diet (LCKD) in overweight and obese patients with type 2 diabetes over 16 weeks. Specifically, we wanted to learn the diet's effects on glycemia and diabetes medication use in outpatients who prepared (or bought) their own meals. In a previous article, we reported the results observed in 7 individuals [10]; this report includes data from those 7 individuals along with data from additional participants enrolled subsequently.

## Methods

### Participants

Participants were recruited from the Durham Veterans Affairs Medical Center (VAMC) outpatient clinics. Inclusion criteria were age 35–75 years; body mass index (BMI) >25 kg/m^2^; and fasting serum glucose >125 mg/dL or hemoglobin A_1c _>6.5% without medications, or treatment with oral hypoglycemic agents (OHA) and/or insulin. Exclusion criteria were evidence of renal insufficiency, liver disease, or unstable cardiovascular disease by history, physical examination, and laboratory tests. All participants provided written informed consent approved by the institutional review board. No monetary incentives were provided.

### Intervention

At the first visit, participants were instructed how to follow the LCKD as individuals or in small groups, with an initial goal of ≤20 g carbohydrate per day. Participants were taught the specific types and amounts of foods they could eat, as well as foods to avoid. Initially, participants were allowed unlimited amounts of meats, poultry, fish, shellfish, and eggs; 2 cups of salad vegetables per day; 1 cup of low-carbohydrate vegetables per day; 4 ounces of hard cheese; and limited amounts of cream, avocado, olives, and lemon juice. Fats and oils were not restricted except that intake of *trans *fats was to be minimized. Participants were provided a 3-page handout and a handbook [11] detailing these recommendations. Participants prepared or bought all of their own meals and snacks following these guidelines.

In addition, on the day the diet was initiated, diabetes medications were reduced – generally, insulin doses were halved, and sulfonylurea doses were halved or discontinued. Due to the possible diuretic effects of the diet soon after initiation, diuretic medications were discontinued if of low dosage (up to 25 mg of hydrochlorothiazide or 20 mg of furosemide) or halved if of higher dosage. Participants were also instructed to take a standard multivitamin and drink 6–8 glasses of water daily, and were encouraged to exercise aerobically for 30 minutes at least three times per week.

Participants returned every other week for 16 weeks for further diet counseling and medication adjustment. When a participant neared half the weight loss goal or experienced cravings, he or she was advised to increase carbohydrate intake by approximately 5 g per day each week as long as weight loss continued. Participants could choose 5 g carbohydrate portions from one of the following foods each week: salad vegetables, low-carbohydrate vegetables, hard or soft cheese, nuts, or low-carbohydrate snacks. Diabetes medication adjustment was based on twice daily glucometer readings and hypoglycemic episodes, while diuretic and other anti-hypertensive medication adjustments were based on orthostatic symptoms, blood pressure, and lower extremity edema.

### Measurements

Participants completed take-home food records (4 consecutive days, including a weekend) collected at baseline and at weeks 2, 8, and 16 during the study. Participants were given handouts with examples of how to complete the records. A registered dietician analyzed the food records using a nutrition software program (Food Processor SQL, ESHA Research, Inc., Salem, OR).

The following measurements were made every other week: anthropometric and vital sign measurements; urine testing for ketones; and assessment for hypoglycemic episodes and other symptomatic side effects. Weight was measured on a standardized digital scale while the participant was wearing light clothes and shoes were removed. Skinfold thickness was measured at 4 sites – the average of 2 measurements at each site was entered into an equation to calculate percent body fat [12]. Waist circumference was measured at the midpoint between the inferior rib and the iliac crest using an inelastic tape; 2 measurements were averaged in the analysis. Blood pressure and heart rate were measured after the participant had been seated quietly without talking for 3 minutes. Certified laboratory technicians assessed urine ketones from a fresh specimen using the following semi-quantitative scale: none, trace (up to 0.9 mmol/L [5 mg/dL]), small (0.9–6.9 mmol/L [5–40 mg/dL]), moderate (6.9–13.8 mmol/L [40–80 mg/dL]), large80 (13.8–27.5 mmol/L [80–160 mg/dL]), large160 (>27.5 mmol/L [160 mg/dL]). Hypoglycemic episodes and symptomatic side effects were assessed by direct questioning of the participant and by self-administered questionnaires.

Blood specimens were obtained at weeks 0, 8, and 16 after the participant had fasted overnight. The following serum tests were performed in the hospital laboratory using standardized methods: complete blood count, chemistry panel, lipid panel, thyroid-stimulating hormone, and uric acid. A non-fasting specimen was also drawn at weeks 4 and 12 to monitor electrolytes and kidney function.

The primary outcome was the change from baseline to week 16 in hemoglobin A_1c_. Changes in all variables were analyzed by the paired t-test or Wilcoxon signed-ranks test, as appropriate. Linear regression analysis was used to examine predictors of change in hemoglobin A_1c_. A p value of 0.05 or less was considered statistically significant. Statistical analysis was performed using SAS version 8.02 (SAS Institute, Cary, NC).

## Results

Of the 28 participants enrolled in the study, 21 completed the 16 weeks of follow-up. Reasons for discontinuing the study included unable to adhere to study meetings and unable to adhere to the diet; no participant reported discontinuing as a result of adverse effects associated with the intervention. All but one of the 21 participants were men; 62% (n = 13) were Caucasian, 38% (n = 8) were African-American (Table 1). The mean age was 56.0 ± 7.9 years.

**Table 1:** Baseline characteristics (n = 21)

**Characteristic**	**Summary**
Age, years, mean (SD)	56.0 (7.9)
Gender, male, n (%)	20 (95%)
Race, White, n (%)	13 (62%)
African-American, n (%)	8 (38%)
Weight, kg, mean (SD)	131.4 (18.3)
BMI, kg/m^2^, mean (SD)	42.2 (5.8)

Adequate food records were available for analysis in a proportion of participants at each of the 4 timepoints (Table 2). Participants completed food records at a mean of 2.5 and a median of 3 timepoints. In general, comparing baseline to subsequent timepoints, mean carbohydrate intake decreased substantially and energy intake decreased moderately while protein and fat intake remained fairly constant.

**Table 2:** Diet composition

**Nutrient**	**Week 0** ***Mean (SD)***	**Week 2** ***Mean (SD)***	**Week 8** ***Mean (SD)***	**Week 16** ***Mean (SD)***
n	14	15	15	8
Carbohydrate, g	204.4 (118.4)	44.6 (27.4)	44.0 (29.1)	33.8 (24.6)
Protein, g	95.8 (23.9)	111.7 (38.6)	114.8 (57.0)	98.5 (52.5)
Fat, g	95.5 (27.3)	95.1 (47.2)	106.6 (47.6)	93.5 (63.7)
Energy, kcal	2031.5 (521.4)	1515.5 (587.2)	1603.4 (713.0)	1418.7 (756.9)

From baseline to week 16, the mean body weight decreased significantly from 131.4 ± 18.3 kg to 122.7 ± 18.9 kg, BMI decreased from 42.2 ± 5.8 kg/m^2 ^to 39.4 ± 6.0 kg/m^2^, and waist circumference from 130.0 ± 10.5 cm to 123.3 ± 11.3 cm (Table 3). The percent change in body weight was -6.6%. The mean percent body fat decreased from 40.4 ± 5.8% to 37.0 ± 6.0%. Systolic and diastolic blood pressures did not change significantly over the 16 weeks. The mean heart rate decreased from 81.2 ± 12.9 beats per minute to 74.6 ± 14.0 beats per minute (p = 0.01).

**Table 3:** Anthropometric and vital sign measurements (n = 21)

**Measurement**	**Week 0** ***Mean (SD)***	**Week 16** ***Mean (SD)***	**Change** ***%***	p value*
Body weight, kg	131.4 (18.3)	122.7 (18.9)	-6.6	<0.001
Body mass index, kg/m^2^	42.2 (5.8)	39.4 (6.0)	-6.6	<0.001
Waist circumference, cm	130.0 (10.5)	123.3 (11.3)	-5.2	<0.001
Percent body fat, %	40.4 (5.8)	37.0 (6.0)	-8.4	<0.001
Systolic blood pressure, mm Hg	135.1 (14.8)	135.4 (17.6)	0.2	0.9
Diastolic blood pressure, mm Hg	79.2 (14.9)	74.1 (13.0)	-6.4	0.1
Heart rate, beats/min	81.2 (12.9)	74.6 (14.0)	-8.1	0.01

*By paired t-test or Wilcoxon signed-ranks test.

Urine ketone data were missing in a median of 4 participants (range 0–8) at any given visit. The proportion of participants with a urine ketone reading greater than trace was 1 of 17 participants at baseline, 5 of 17 participants at week 2, and similar frequencies at subsequent visits until week 14 when 2 of 18 participants had readings greater than trace and week 16 when 2 of 21 participants had readings greater than trace. During the study, only 27 of 151 urine ketone measurements were greater than trace, with one participant accounting for all 7 occurrences of the highest urine ketone reading (large160).

In regard to serum measurements, the mean fasting glucose decreased by 17% from 9.08 ± 4.09 mmol/L at baseline to 7.57 ± 2.63 mmol/L at week 16 (p = 0.04) (Table 4). Serum sodium and chloride levels increased significantly, but only by 1% and 3%, respectively. Uric acid level decreased by 10% (p = 0.01). Serum triglyceride decreased 42% from 2.69 ± 2.87 mmol/L to 1.57 ± 1.38 mmol/L (p = 0.001). Increases occurred in both high-density lipoprotein (HDL) cholesterol (8%) and low-density lipoprotein (LDL) cholesterol (10%) but these changes were of borderline statistical significance (p = 0.08 and p = 0.1, respectively). The following blood tests did not change significantly: total cholesterol, potassium, bicarbonate, urea nitrogen, creatinine, calcium, thyroid-stimulating hormone, and hemoglobin.

**Table 4:** Serum test results (n = 21)

**Measurement**	**Week 0** ***Mean (SD)***	**Week 16** ***Mean (SD)***	**Change** ***%***	p value*
Hemoglobin A_1c_, %	7.5 (1.4)	6.3 (1.0)	-16.0	<0.001
Glucose, mmol/L	9.08 (4.09)	7.57 (2.63)	-16.6	0.04
Total cholesterol^†^, mmol/L	4.61 (1.40)	4.54 (1.26)	-1.5	0.7
Triglyceride^†^, mmol/L	2.69 (2.87)	1.57 (1.38)	-41.6	0.001
HDL-C^†^, mmol/L	0.92 (0.20)	0.99 (0.22)	7.6	0.08
LDL-C^†^, mmol/L	2.51 (0.64)	2.77 (0.89)	10.4	0.1
Sodium, mmol/L	138.2 (3.4)	140.2 (3.4)	1.4	0.02
Potassium, mmol/L	4.2 (0.4)	4.2 (0.3)	0	0.2
Chloride, mmol/L	101.0 (3.4)	103.8 (2.8)	2.8	0.001
Bicarbonate, mmol/L	28.8 (2.0)	28.2 (2.5)	-2.1	0.3
Urea nitrogen, mg/dL	5.90 (1.90)	6.27 (1.81)	6.3	0.3
Creatinine, μmol/L	82.8 (20.4)	79.9 (19.1)	-3.5	0.3
Calcium^†^, mmol/L	2.32 (0.11)	2.33 (0.08)	0.4	0.8
Uric acid^†^, μmol/L	403.5 (94.6)	352.1 (85.4)	-10.3	0.01
TSH^†^, mIU/L	1.6 (1.0)	1.4 (0.7)	-12.7	0.2
Hemoglobin, g/L	142 (11)	141 (10)	-0.7	0.8

Legend: TSH = thyroid-stimulating hormone, HDL-C = high-density lipoprotein cholesterol, LDL-C = low-density lipoprotein cholesterol.

* By paired t-test or Wilcoxon signed-ranks test.

^† ^Serum calcium, TSH, total cholesterol, triglyceride, and HDL-C results are based on n of 20 because one participant did not have these tests at baseline. Uric acid is based on n of 19. LDL-C is based on n of 17 because triglyceride levels were too high at baseline to calculate LDL-C in 3 participants.

**Figure 1:**
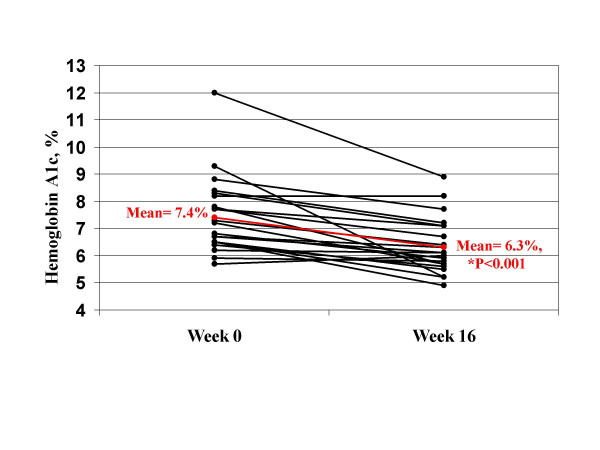
**Hemoglobin A1c for each participant**. *Red line is the group mean. P value is for the mean change from baseline.

The primary outcome, hemoglobin A_1c_, decreased from 7.5 ± 1.4% at baseline to 6.3 ± 1.0% at week 16 (p < 0.001), a 1.2% absolute decrease and a 16% relative decrease (Table 4). All but two participants (n = 19 or 90%) had a decrease in hemoglobin A_1c _(Figure 1). The absolute decrease in hemoglobin A_1c _was at least 1.0% in 11 (52%) participants. The relative decrease in hemoglobin A_1c _from baseline was greater than 10% in 14 (67%) participants, and greater than 20% in 6 (29%) participants. In regression analyses, the change in hemoglobin A_1c _was not predicted by the change in body weight, waist circumference, or percent body fat at 16 weeks (all p > 0.05).

The improvement in glycemic control occurred while medications for diabetes were discontinued or reduced in most participants (Table 5). During the study, hypertension and hyperlipidemia medication doses were not increased from baseline nor were new agents added, except in 3 individuals. No serious adverse effects related to the diet occurred. One participant had a hypoglycemic episode requiring assistance from emergency services after he skipped a meal but the episode was aborted without need for transportation to the emergency room or hospitalization.

**Table 5:** Diabetes medication changes

**Participant**	**Week 0** ***Total daily dose***	**Week 16** ***Total daily dose***
**Participants with diabetes medications discontinued (n = 7 of 21)**		
5	glipizide 10 mg metformin 1000 mg	none
6	glipizide 20 mg metformin 1500 mg	none
7	metformin 2000 mg rosiglitazone 8 mg	none
9	metformin 1000 mg	none
15	metformin 1000 mg	none
22	metformin 1000 mg	none
24	metformin 1000 mg	none
**Participants with diabetes medications reduced (n = 10 of 21)**		
3	70/30 insulin 50 units metformin 1000 mg	metformin 1000 mg
11	metformin 2000 mg glyburide 20 mg	metformin 2000 mg
16	metformin 2000 mg pioglitazone 45 mg glipizide 20 mg	metformin 2000 mg
21	metformin 1500 mg pioglitazone 30 mg	metformin 1000 mg
8	NPH 145 units metformin 1000 mg	NPH 25 units metformin 1000 mg
13	70/30 insulin 70 units metformin 2550 mg	70/30 insulin 35 units metformin 2550 mg
23	70/30 insulin 110 units pioglitazone 45 mg	70/30 insulin 80 units pioglitazone 45 mg metformin 1000 mg
25	NPH 70 units, R 30 units metformin 2000 mg pioglitazone 45 mg	NPH 8 units metformin 2000 mg pioglitazone 45 mg
27	70/30 insulin 86 units metformin 2000 mg	70/30 insulin 18 units metformin 2000 mg
28	NPH insulin 110 units lispro insulin 90 units glipizide 20 mg	NPH insulin 30 units glipizide 20 mg
**Participants with diabetes medications unchanged (n = 4 of 21)**		
1	none	none
2	metformin 1700 mg	metformin 1700 mg
10	none	none
26	metformin 2000 mg	metformin 2000 mg

## Discussion

In this single-arm, 4-month diet intervention, an LCKD resulted in significant improvement of glycemia, as measured by fasting glucose and hemoglobin A_1c_, in patients with type 2 diabetes. More importantly, this improvement was observed while diabetes medications were reduced or discontinued in 17 of the 21 participants, and were not changed in the remaining 4 participants. Participants also experienced reductions in body weight, waist circumference, and percent body fat but these improvements were moderate and did not predict the change in hemoglobin A_1c _in regression analyses.

Several recent studies indicate that a low-carbohydrate diet is effective at improving glycemia. A few studies have shown that in non-diabetic individuals, low-carbohydrate diets were more effective than higher carbohydrate diets at improving fasting serum glucose [13,14] and insulin [6,14-16], and at improving insulin sensitivity as measured by the homeostasis model [6]. One of these studies also included diabetic patients and noted a comparative improvement in hemoglobin A_1c _after 6 months (low fat diet: 0.0 ± 1.0%; low carbohydrate diet: -0.6 ± 1.2%, p = 0.06) [6] and 12 months (low fat diet: -0.1 ± 1.6%; low carbohydrate diet: -0.7 ± 1.0%, p = 0.019) duration [5]. In a 5-week crossover feeding study, 8 men with type 2 diabetes had greater improvement in fasting glucose, 24-hour glucose area-under-the-curve (AUC), 24-hour insulin AUC, and glycohemoglobin while on the low-carbohydrate diet than when on a eucaloric low-fat diet [7]. In a 14-day inpatient feeding study, 10 participants with type 2 diabetes experienced improvements in hemoglobin A_1c _and insulin sensitivity as measured by the euglycemic hyperinsulinemic clamp method [8]. Hemoglobin A_1c _also improved in an outpatient study of 16 participants who followed a 20% carbohydrate diet for 24 weeks [9].

Similar to our results, three studies noted that diabetes medications were reduced in some participants[6,8,9], although details were provided in only one study. We also discontinued diuretic medications during diet initiation because of concern for additional diuresis incurred by the diet. This concern was based on the theoretical effects of the diet [17], observed effects of the diet on body water by bioelectric impedance [18], and practical experience with the diet [19]. Until we learn more about using low carbohydrate diets, medical monitoring for hypoglycemia, dehydration, and electrolyte abnormalities is imperative in patients taking diabetes or diuretic medications.

While body weight decreased significantly (-8.5 kg) in these 21 diabetic participants, the mean weight loss was less compared with what we observed in the LCKD participants of an earlier trial (-12.0 kg) [18]. Given that the diabetic participants had a higher baseline mean weight than the LCKD participants of our previous trial (131 kg vs. 97 kg), this translates into an even more dramatic disparity in percent change in body weight (-6.6% vs. -12.9%). This lesser weight loss might result from several factors. First, in the current study, most of the participants were taking insulin and/or oral hypoglycemic agents that are known to induce weight gain[20,21] Second, these same agents, particularly insulin, inhibit ketosis, which is strived for in the earliest phases of the LCKD; while it remains unclear whether ketones actually play a role in weight loss on the LCKD, previous research in non-diabetic patients has shown a positive correlation between level of ketonuria and weight loss success [22]. Lastly, compared with our previous study the participants in the current study had more comorbid illness, lower socioeconomic status, and a shorter duration of follow-up (16 weeks versus 24 weeks), all of which are associated with reduced success on any weight loss program [23].

The main limitations of our study are its small sample size, short duration, and lack of control group. That the main outcome, hemoglobin A_1c_, improved significantly despite the small sample size and short duration of follow-up speaks to the dramatic and consistent effect of the LCKD on glycemia. For other effects, however, such as the rises in serum LDL and HDL cholesterol, the small sample size might be the reason statistical significance was not reached. Future studies of larger samples and containing a control group are needed to better address questions about the effect of the LCKD on serum lipids in patients with type 2 diabetes.

## Conclusion

In summary, the LCKD had positive effects on body weight, waist measurement, serum triglycerides, and glycemic control in a cohort of 21 participants with type 2 diabetes. Most impressive is that improvement in hemoglobin A_1c _was observed despite a small sample size and short duration of follow-up, and this improvement in glycemic control occurred while diabetes medications were reduced substantially in many participants. Future research must further examine the optimal medication adjustments, particularly for diabetes and diuretic agents, in order to avoid possible complications of hypoglycemia and dehydration. Because the LCKD can be very effective at lowering blood glucose, patients on diabetes medication who use this diet should be under close medical supervision or capable of adjusting their medication.

## Competing interests

Dr. Vernon has held a consulting relationship with Atkins Nutritionals, Inc.

## Authors' contributions

WY conceived, designed, and coordinated the study; participated in data collection; performed statistical analysis; and drafted the manuscript. MF assisted with study design, performed data collection, and helped to draft the manuscript. AC analyzed the food records. MV assisted with study/intervention design and safety monitoring. EW participated in the conception and design of the study, and assisted with the statistical analysis. All authors read and approved the final manuscript.
